# Effect of Internet-Based Cognitive Apprenticeship Model (*i*-CAM) on Statistics Learning among Postgraduate Students

**DOI:** 10.1371/journal.pone.0129938

**Published:** 2015-07-01

**Authors:** Farzaneh Saadati, Rohani Ahmad Tarmizi, Ahmad Fauzi Mohd Ayub, Kamariah Abu Bakar

**Affiliations:** 1 Institute for Mathematical Research, Universiti Putra Malaysia, Serdang, Selangor, Malaysia; 2 Department of Science, Jundi-Shapur University of Technology, Dezful, Iran; 3 Faculty of Educational Studies, Universiti Putra Malaysia, Serdang, Selangor, Malaysia; University of Westminster, UNITED KINGDOM

## Abstract

Because students’ ability to use statistics, which is mathematical in nature, is one of the concerns of educators, embedding within an e-learning system the pedagogical characteristics of learning is ‘value added’ because it facilitates the conventional method of learning mathematics. Many researchers emphasize the effectiveness of cognitive apprenticeship in learning and problem solving in the workplace. In a cognitive apprenticeship learning model, skills are learned within a community of practitioners through observation of modelling and then practice plus coaching. This study utilized an internet-based Cognitive Apprenticeship Model (*i*-CAM) in three phases and evaluated its effectiveness for improving statistics problem-solving performance among postgraduate students. The results showed that, when compared to the conventional mathematics learning model, the *i*-CAM could significantly promote students’ problem-solving performance at the end of each phase. In addition, the combination of the differences in students' test scores were considered to be statistically significant after controlling for the pre-test scores. The findings conveyed in this paper confirmed the considerable value of *i*-CAM in the improvement of statistics learning for non-specialized postgraduate students.

## Introduction

Supporting students, especially non-specialised students, in a course that is mathematical in nature, is always a concern of instructors [1]. According to Jaki and Autin [2], the standard approach to teaching statistics is usually teacher-centred, which emphasizes a particular learning style with students. With this method, the lecturer tries to impose his/her knowledge upon the students, who more often than not lose the connections of lessons when dealing with various statistical methods. On the other hand, the effort to engage graduate students in a genuine learning experience and the application of statistics in original research is an approach suggested for the teaching and learning of educational statistics [3]. Over recent years, mathematics educators and statisticians have devoted large segments of their careers to an effort to improve pedagogical techniques and educational materials in statistics [4]. In line with emergent pedagogical insights, this approach includes a number of paradigm shifts in the educational field—a shift from behaviourism to cognitivism [5] and from individual learning to collaborative learning [6]. These shifts entail moving from ‘passive’ learning to ‘active’ learning through a change of learning perspective from teacher-centred learning to learner-centred learning [7]. Educational researchers speculate that active learning in collaborative groups of students can increase individual learning performance [8]. Accordingly, collaborative learning as a method of student-centred active learning can engage graduate students in a genuine learning experience and help them to better understand how to apply statistics [7].

Many instructional tools—physical tools as well as electronic devices—have been used for many years to help teach mathematics. Sometimes, has served as a way to creatively depict mathematical ideas, progressing in accordance with the evolution of mathematics itself; while at other times, technology has entered mathematics, notably from science and commerce [9]. Since the mid-1990s, computer-mediated communication has been widely implemented for online education as the main delivery method in blended learning and also as a support tool. According to Roberts et al. [9], technology has opened a window in which mathematics education might enter into a new epistemological domain, where knowledge can become personal and communal, and connective and explorative mathematical knowledge would become more accessible. A Learning Management System (LMS) is one of the latest technological systems employed by almost all universities throughout the world. Researchers have estimated that technologies of the future will be used more, not less, within the education system, including for the purpose of engaging learners of varying abilities [10].

Embedding within e-learning systems the pedagogical characteristics of learning, in order to facilitate a conventional way of learning, is value added. Many different LMSs (such as Moodle) are used in the teaching and learning process, but there is a lack of innovation to adopt effective instructional approaches on these learning platforms. Hence, the need exists to begin using these technological platforms or LMSs in new ways in order to advance beyond what is currently possible in the classroom. In addition, the possibility of instruction using online learning interactions as a hybrid or blended-learning approach will be deemed necessary to support face-to-face courses or lecture-delivered. However, this training model, which is considered for implementation in the physical workplace, can be structured to be employed entirely virtually, especially to improve students’ learning performance. This study was undertaken to identify the impact of the Moodle-based Cognitive Apprenticeship Model (CAM) on learning patterns and on students’ learning performance using a blended-learning mode for postgraduate students taking a course in educational statistics.

### Cognitive Apprenticeship Model

According to Brown et al. [11], there is a gap between school learning and real-life application. Resnick [12] suggested ‘Bridging Apprenticeships’ as a way to make explicit the tacit knowledge. Formal learning as a learning component takes place outside of the workplace, such as in university classrooms. However, Foley [13] identified another part of learning to be informal learning in a workplace. It involves what will be learned through experiences on the job, where a practitioner’s act will reflect on an action and then learning from that reflection plans a new action [13]. This is known as apprenticeship, which is considered to be an inherently social learning approach. It has a long history of helping novices to become experts in various fields, such as midwifery, construction, and law [14]. The central aim of apprenticeship is the concept in which experienced people assist less-skilled ones by providing structures and examples to support the goals to be achieved. This method of teaching through modelling, coaching, and fading is the common form of learning for many learners [11].

Situated learning [15] or situated cognition [16] is associated with notion of learning through social development. Learning as a process of social participation involves participation in a “community of practice” [17]. Furthermore, learning would be meaningful and significant if it is situated or embedded within an authentic activity, community, context, social engagement, or culture with a real-world learning context [11].Cognitive apprenticeship [11, 18] is a strategy for creating learning environments that incorporate many of the salient features of situated cognition. According to Oriol et al. [19], cognitive apprenticeship strategies recommend a robust and rigorous approach for teaching complex problem-solving skills and developing vital experiences contained in a discipline. Furthermore, Brown et al. [11] proposed the Cognitive Apprenticeship Theory, which is based on Vygotsky’s Zone of Proximal Development (ZPD), in 1978. The ZPD is supposed to be a dynamic region, which is just beyond the present level of learner's ability. This region or ZPD will move with learners' development as they achieve new understanding and skills.This model endeavours to make visible for novices what is an invisible part of the expert’s thinking [18, 20]. Some cognitive apprenticeship models have been constructed to enhance learning and instruction [11, 21, 22]. In cognitive apprenticeship learning, the experts model the skills and the learners observe that modelling. The learners then practice the skills supported by coaching from the experts [23]. The model that Brown et al. [11] proposed listed six major steps as follows:

1. Modelling: Experts try to demonstrate and explain their own way of thinking for learners to monitor and understand.
2. Coaching: In this step, learners practise those observed methods on their own, while the experts give advice and even correct them.
3. Scaffolding: Through increasing the complexity of the problems, the level of assistance decreases as the learners’ progress increases; therefore, experts progressively help the learners until they can independently accomplish a task.
4. Articulation: In this step, learners are given opportunities to explain and articulate their own way of thinking.
5. Reflection: Learners can compare their own thoughts and ways with those of the experts and their peers.
6. Exploration: Learners can manipulate and discover the learned knowledge or skills in order to promote their accurate understanding.

The main intent of cognitive apprenticeship, which has some similarity to ZPD, is to engage the learner in meaningful, constructive activities that encourage augmentation and preparation of new skills and conceptions [24, 25]. In fact, the introduction of multiple methods in this model helps the learner accomplish a task through different degrees of skill, helps the learner recognize that no one is an embodiment of expertise, and encourages learners to understand that learning is a continuing process [17].

## Internet-Based Cognitive Apprenticeship Model

Vygotsky’s theory stressed social interactions among teachers and peers, while Collins [26] emphasized that technology can play a key role in creating performance supports and learning environments. Alger and Kopcha [20] showed that an LMS has the potential to be used as an academic learning portal to create a dynamic and constructive environment. Moreover, the function of cognitive apprenticeship—using online exhibition of complex concepts and applications—enables the instructors (the experts) and the students (the apprentices) to collaborate and interact in a virtual setting while they effectively take part in the learning process [27]. Such an environment can support students as they perform professional work and as they focus on achieving course competencies throughout the master’s program—especially for adult learners who are struggling to balance attendance at face-to-face classes, their personal responsibilities, and their employment [19]. Among all the features of LMSs, discussion forums represent a specific role in online communication. They allow instructors and experts to act as facilitators [28], while the students experience virtual face-to-face sessions that are controlled by the facilitators. Accordingly, this demands active participation with stimulating activities to encourage the continued participation in the course activities [29–31].

This study adopted the generic model of Brown et al. [11] and the web-based cognitive apprenticeship model of Liu [32] to design a three-phase model based on learners’ needs and on characteristics of forums in an LMS. This model is considered to be an Internet-based cognitive apprenticeship model (*i*-CAM) to help postgraduate students learn statistics, while a discussion forum serves as the medium platform.

### Handling Phase

The first phase was an intervention phase, the main aim of which was to provide students with a broad contextual understanding within a constructivist learning environment. The intervention and activities that occurred during this phase were completely organized and guided by a monitor, while students were asked to follow and visit the portal. This phase of the treatment consisted of a series of activities designed to be consistent with the characteristics of modelling and coaching. The specific goal of having expert monitors demonstrate the activities was to help the students understand this online learning process.

During the first phase of intervention in the experiment group, the activities were deliberately ordered and sequenced throughout the modelling and coaching to improve the students’ ability to learn statistics and solve problems. This pattern was followed with conceptual knowledge by sharing related resources and special procedural knowledge, focusing on the statistics problem-solving procedures. For instance, the monitor shared a problem scenario on the forum and described the five steps of problem-solving. Students were supposed to follow all steps, and if they had any questions and difficulties, they could post them as comments.

### Supporting Phase

At this stage, students received, on the learning forum, a shared set of scenarios involving statistics problems. The monitor began by posting some questions and sufficient hints regarding how to solve the problem for each step of the problem-solving process. The researcher scaffolded the learners to achieve the specific solution in each scenario. The scaffolding continued in a similar way, and gradually the number of hints or the amount of scaffold was reduced and subsequently stopped. In addition, consistent with the CAM approach, at this stage of the interaction phase, students were systematically encouraged to take part in the articulation and reflection processes.

Throughout the next stage of the forum practices, the students were encouraged to articulate their thoughts on how to solve a statistics problem as well as to indicate the difficulties that may have occurred during their performance. Moreover, since the activities in this stage were conducted in groups, via a cooperative learning approach, students actively interacted with each other to reflect on the work they had already performed and analysed. This allowed them to compare what they know with what others know.

### Self-Exploring Phase

The self-exploring phase was a withdrawal phase, since there were no specified interventions by the monitor. Throughout this phase, the monitor withheld involvement while the students continued their online activities. In this phase, the monitor provided conditions that could push the learners into manipulating and exploring what they learned to support their true understanding or explain their troubleshooting, if any. In this phase too, the monitor did not have any interactions with the learners, but the forum was active, with two or three good-standing volunteers continuing the discussion there. It was, therefore, possible for learners to share some supportive materials, the completed assignments, and helpful links.

The participants were asked to discuss the challenges that they encountered while doing their assignments. The forum allowed them to share their solutions and correct each other. This practice enabled them to be independent problem solvers as well. In cases in which some assistance was needed, the good-standing and master volunteers provided coaching throughout the discussions. During this period, the monitor gave little assistance, except in emergency cases for safety purposes in order to control students’ misunderstandings.

## Research Questions

In this study, the above-mentioned activities were designed based on *i*-CAM and were added as an instructional model in a blended-learning course. This experiment specifically examined the effects of *i*-CAM for postgraduate students in the learning of statistics as compared to the effects of generic blended learning. This study is guided by the following research questions:

Q 1) What are the effects of each phase of *i*-CAM in terms of improved student learning of statistics as compared to performance outcomes from conventional learning?

Q 2) Are there any significant differences in the means of the students’ statistics learning across Tests I, II, and III, between the *i*-CAM and the generic blended groups, while controlling for the scores on the tests administered before the program?

## Methodology

### Ethics Statements

This study was reviewed and approved by the Research Committee of Laboratory of Ethnomathematics and Didactics, INSPEM, in terms of ethical issues. Written consent was obtained from each of the participants after the process of the study was explained clearly before starting the experiment.

### Research Design

The study utilized the quasi-experimental design to collect sufficient data to answer the research questions. While the study attempted to verify the effectiveness of the *i*-CAM versus the generic blended-learning model among target learners, data were collected from two different groups of students across two semesters in order to assess and evaluate the supporting activities. To control the intervention between these two groups of students during the portal access learning, the researcher had to select the students from two different semesters. This quasi-experiment was used as Shadish et. al. [33] with two groups in which each group consisting of a different cohort of students during two semesters. It was useful in situations in which as one group finishes the course, their places are taken by another group. In addition, the two groups of students had similar characteristics and had followed the requirements of the course on Educational Statistics.

A total of 53 postgraduate students enrolled in the Educational Statistics course from the Faculty of Educational Studies participated in this study. Two groups of students were selected from two different semesters as two cohorts, where 27 students were in the treatment group and 26 students as a following cohort were in the control group. The first group of students was selected as the treatment group named the *i*-CAM group and given an access account, which was deactivated at the end of the semester before registering the new group of students as the control group or the conventional group.

The learning strategy based on the *i*-CAM played an important role in facilitating students’ participation in the Educational statistics Course. The course incorporated one 3-hour face-to-face lecture each week for 12 weeks. The nature of the course was blended learning, where in addition to the face-to-face lecturer-led instructions organized for three hours per week, students could access a learning portal with a wide source of tutoring materials (e.g. videos, e-books, extra notes, mind maps,…) and communication tools (e.g. Chatroom, Email, Forums).

A total of 2 instructors involved in this study, a professional lecturer taught the face-to-face classes and the Internet-based activities administered by an expert as the monitor. Although the both groups in the study used the same textbook. The instructor assigned the course topics into three portions as descriptive statistics, comparing means, and measures of association while controlled the class pace in each topic. The *i*-CAM as an instructional strategy was added in this portal in contrast to the generic and common use of the portal.

### Procedure

The main idea of this experimental study is to evaluate the effectiveness of the *i*-CAM as an instructional strategy against common use of Moodle. At the beginning of the study, both groups have been registered on the portal and took part in a pre-statistical knowledge, as a pre-test in first session of the class. Moreover, in the first face-to-face session of the class, students received a brief overview and introduction of the learning portal. For example, they learnt how to download the lecture handout or get started a discussion.

**Fig 1:**
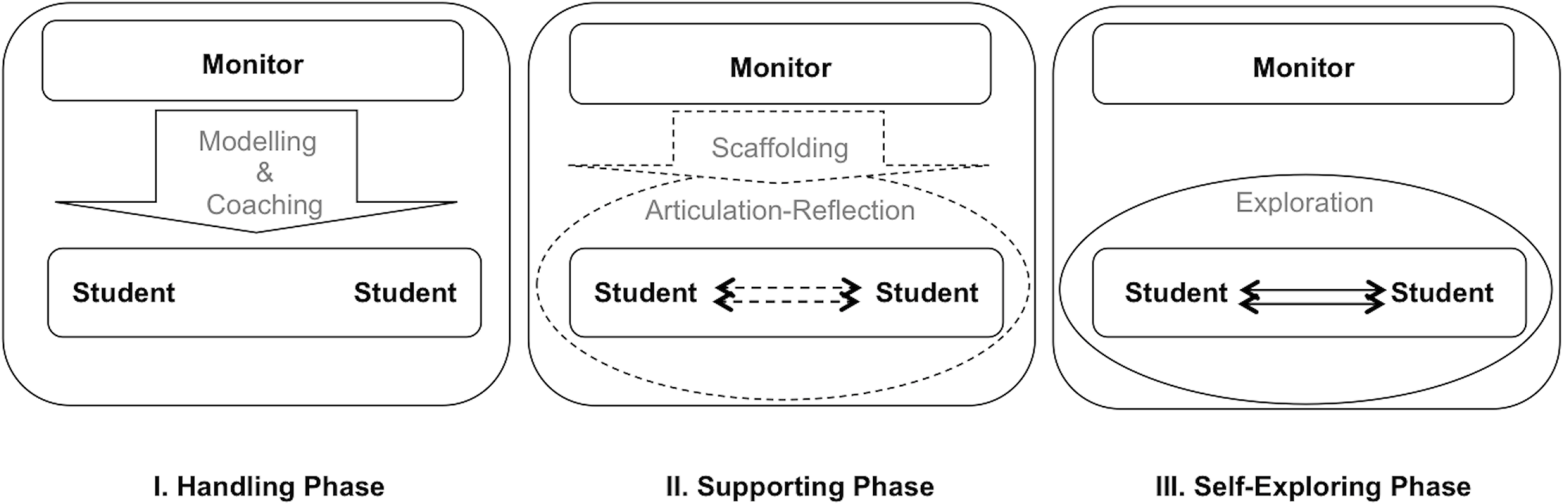
Three-Phase Model of *i*-CAM.

The key point of contrast between the control and experimental group was the method employed on the intervention phases. In the experimental group, the intervention between the monitor and learners was particularly developed by employing the Internet-based Cognitive Apprenticeship Model described as the *i*-CAM (Fig 1).

The conventional group received the same supporting materials and resources on the portal. The students could go to the learning portal and use the learning materials, as well as having discussions with other peers or asking the monitor’s assistance after each face-to-face class.

At the end of each phase, a statistics performance test was administered based on the reviewed topics (descriptive statistics, comparing means, or measures of association), for the both groups of participants.

#### Pre-testing

Pre-testing was utilized to assess the level of basic knowledge among the target students. The standard test included 15 objective-style questions designed by Johnson and Kuennen [34] to assess basic mathematics skills in an introductory statistics course. It was used to measure the basic skills that are needed for successful performance in statistics. This test was administered before starting the instruction to determine the level of the students’ statistics problem-solving skills. A copy of the entire test is published in Johnson and Kuennen [34].

#### Intervention

The intervention was designed in three phases of the *i*-CAM: handling, scaffolding, and self-exploring. Before the intervention, the students received a brief overview of the stages’ activities prior to moving into the *i*-CAM phases. Each phase continued for four weeks, during which students could access the learning portal and interact with the monitor based on defined activities for each phase.

#### Post-testing

Three post-tests were administered to assess the important sub-skills following instruction, and the students’ statistics problem-solving performance was evaluated. These tests were designed parallel to the pre-tests to measure the instructional objectives, which included the statistics problem-solving skills. The items were drawn from the lecturer’s test banks. To establish the test validity, copies of the three tests were sent to four expert statistics lecturers to judge the appropriateness of the items. KR-20 was also computed as a measure of interval consistency. These tests were conducted at the end of each phase. The total scores were considered as post-testing scores for each test. The example items are shown in Appendix 1.

## Results

Four similar tests were designed to measure students’ statistics problem-solving performance. Students took the first test of basic statistics performance with regard to their previous knowledge as a pre-test before starting the treatment. The following tests were conducted at the end of their respective phases of the treatment.

Table 1 shows the means and standard deviations of students’ problem-solving performance statistics on three different topics (Test I, Test II, and Test III) for both the treatment and control groups. The scores of all tests ranged from 0 to 100.The scores of all tests ranged from 0 to 100. S1 Fig showed the descriptive analysis and graphical explanation of the dataset for each of these tests. The entire data set obtained in this study was given in S1 Dataset. Comparing the pre-testing scores gives the idea that before starting the program the students in the control group (M_0_ = 57.12, SD = 26.83) performed better on the basic statistics test than did the students in the treatment group (M_0_ = 55.68, SD = 24.86). The overall mean scores of students’ statistics performance in the treatment group were reported as follows: for Test I (M_1_ = 78.22, SD = 11.01), Test II (M_2_ = 69.72, SD = 13.44), Test III (M_3_ = 58.74, SD = 9.46); while students in the control group achieved the following mean scores: Test I (M_1_ = 67.18, SD = 11.56), Test II (M_2_ = 60.49, SD = 16.81), and Test III (M_3_ = 46.24, SD = 13.13). These results indicated that the treatment group had higher overall mean scores than the control group at the end of each phase of the *i*-CAM.

**Table 1:** Means and Standard Deviations for Problem-Solving Performance.

				Pre-test Adjusted	
Test	Group	Mean	SD	Mean	SE
**Pre-test**	Treatment	55.68	24.86	-	-
	Control	57.12	26.83	-	-
**Test I**	Treatment	78.22	11.01	78.39	1.89
	Control	67.18	11.56	67.91	1.93
**Test II**	Treatment	69.72	13.44	69.89	2.68
	Control	60.49	16.81	59.69	2.73
**Test III**	Treatment	58.74	9.46	58.62	2.01
	Control	46.24	13.13	46.38	2.13

**Fig 2:**
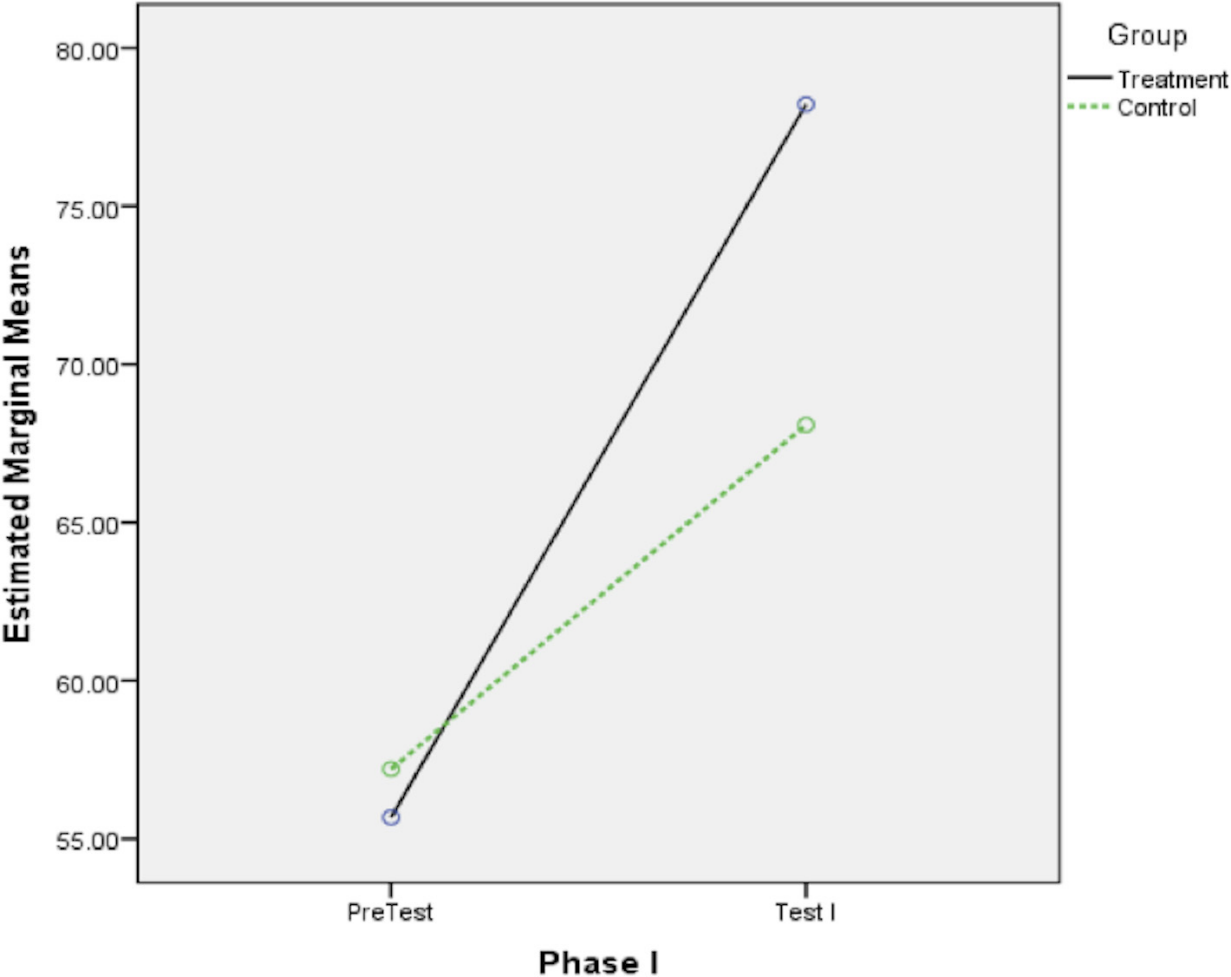
Phase I, Estimated Marginal Means of Statistics Problem-Solving Performance.

The scores for Test I on descriptive statistics for students in the control group increased +10.06 units over the pre-test (M_0_ = 57.12, and M_1_ = 67.18), while in the same tests, students in the treatment group showed +22.54 units improvement for Test I (M_0_ = 55.68, and M_1_ = 78.22). Fig 2 provides a quick visual display of this difference.

**Fig 3:**
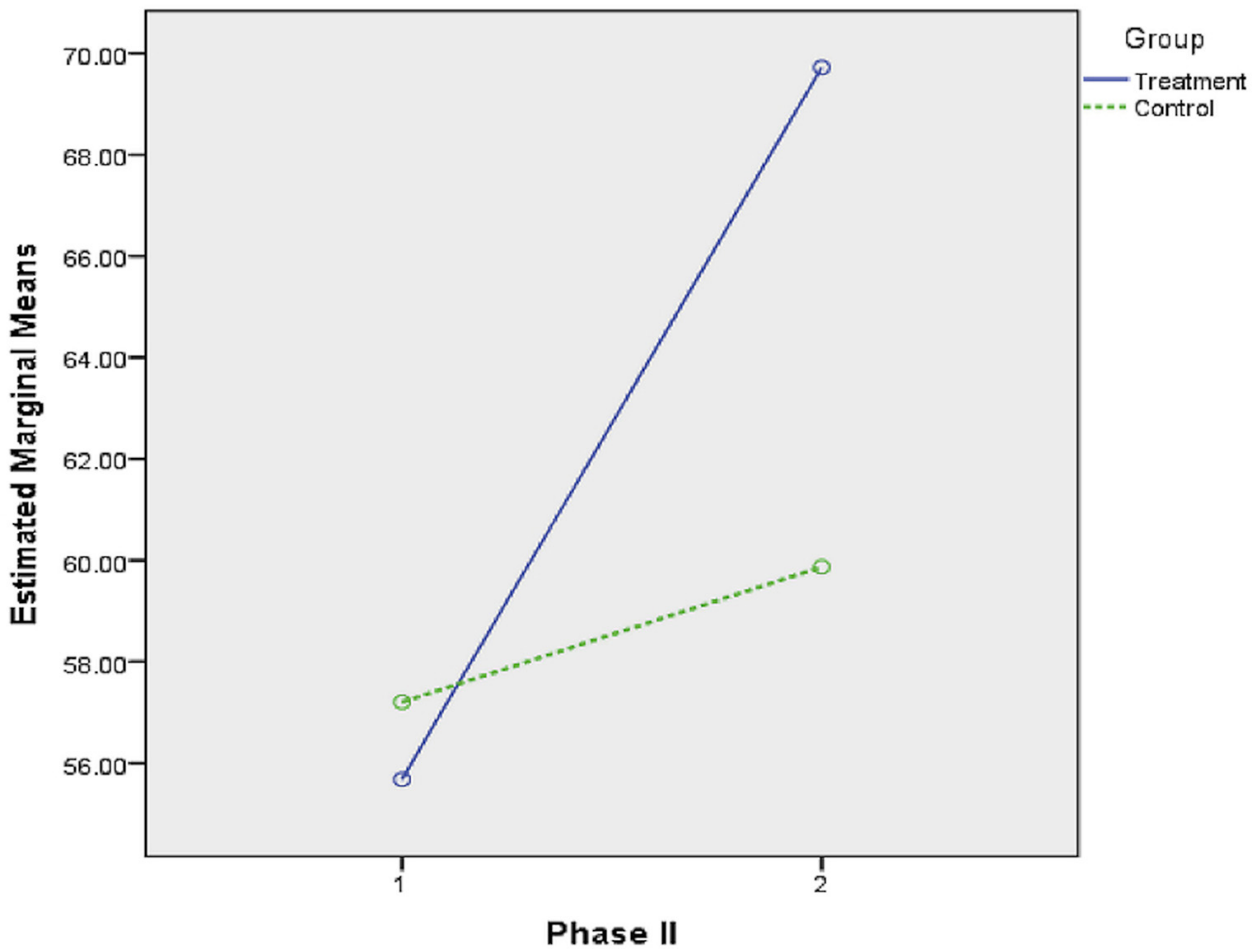
Phase II, Estimated Marginal Means of Statistics Problem-Solving Performance.

Test II was conducted at the end of Phase II of the treatment, which was designed based on scaffolding, articulation, and reflection and was known as the supporting phase. Test II was conducted at the end of the scaffolding phase, and the results showed that students in the control group increased their performance by +3.37 units over the pre-test (M_0_ = 57.12, and M_2_ = 60.49), while at the same time with the same tests, students in the treatment group showed +14.04 units improvement (M_0_ = 55.68, and M_2_ = 69.72). Fig 3 highlights this difference.

**Fig 4:**
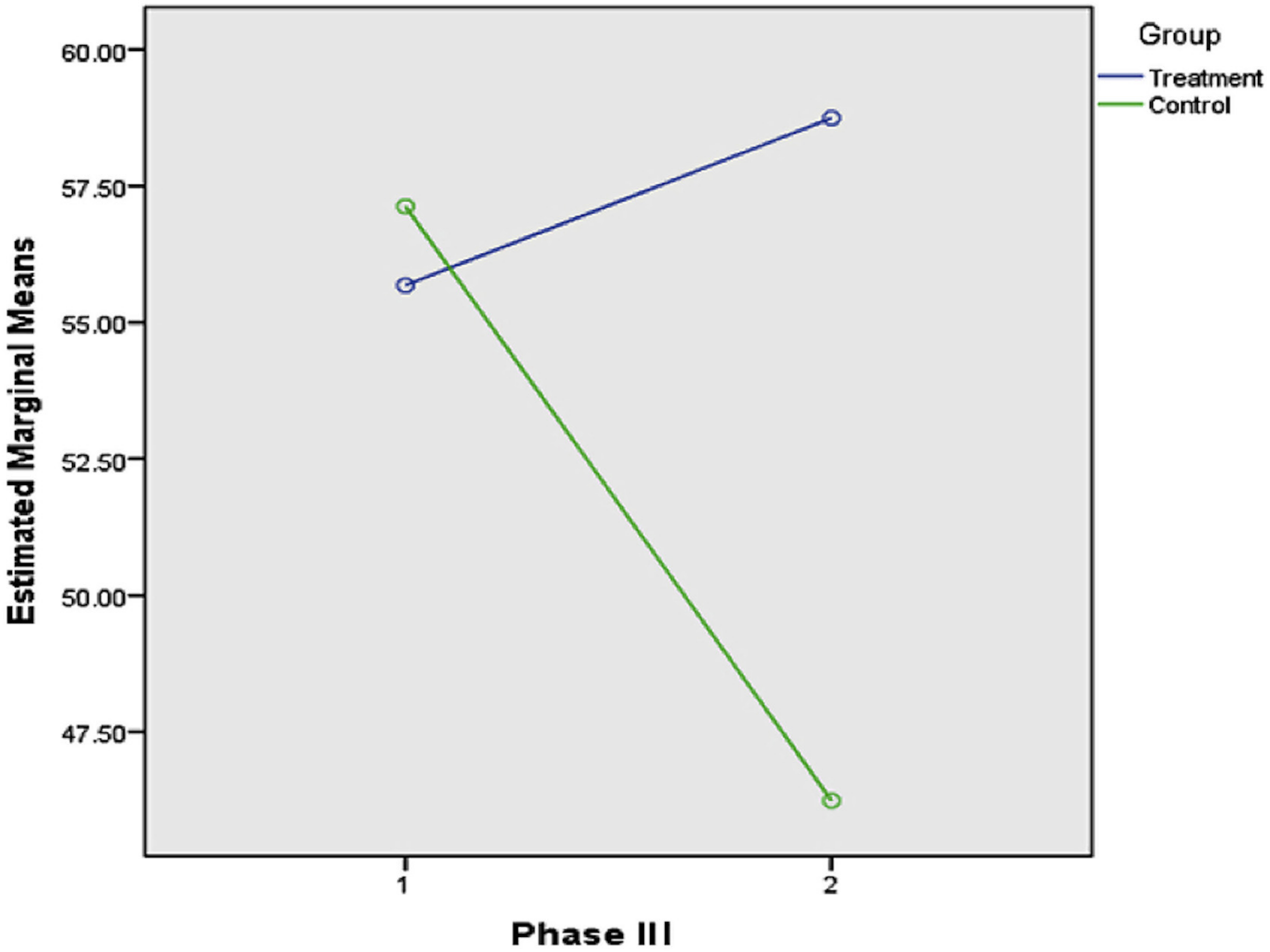
Phase III, Estimated Marginal Means of Statistics Problem-Solving Performance.

Test III was conducted after the last phase of treatment, known as the self-exploring phase, which was a follow-up phase. Actually, there was no difference among activities designed for both groups as well as no interactions between the monitor and the students in the treatment group. The obtained performance scores for this test for the treatment group showed an improvement over the pre-test (M_0_ = 55.68, and M_3_ = 58.74) in comparison to the students in the control group (M_0_ = 57.12, and M_3_ = 46.24). Fig 4 clearly presents this difference.

Three individual ANCOVA were conducted as an appropriate statistical technique to compare the effectiveness of the treatment on students’ statistics performance in each phase of the *i*-CAM. In these statistics tests, students’ scores on the pre-test of basic statistics knowledge were used as the covariates. Preliminary data analysis did not determine any violation of the assumptions of normality, linearity, homogeneity of variances, homogeneity of regression slopes, and reliable measurement of the covariate.

When the effect of the covariate was statistically removed, the adjusted means on Test I of statistics performance were evaluated for the treatment group (M = 78.39, SE = 1.89), and control group (M = 67.91, SE = 1.93). In this case, the results of between-group tests confirmed that after adjusting for pre-test scores, there was a significant difference between these two groups of students, the *i*-CAM group (the treatment group) and the conventional group, F(1, 50) = 15.096, p<0.01. The effect size was considered as a large effect size, with partial eta squared value = 0.23. This indicated that 23% of the variance in Test I scores of statistics performance was explained by their treatment as an independent variable.

The statistical test for evaluating the effectiveness of Phase II demonstrated that the adjusted mean for the statistics performance of the treatment group on Test II was M = 69.89 (SE = 2.68) and the control group was M = 59.69 (SE = 2.73) after removing the effect of the covariate. Consequently, after adjusting for pre-test scores, the students of the *i*-CAM group and the conventional group performed significantly differently in Test II,F(1, 50) = 7.118, p<0.05, with a partial eta squared value equal to 0.125,which was a moderately large effect size.

Table 1 above demonstrated the means of statistics performance scores for Test III and the results of the tests conducted to compare the means between groups after adjusting for pre-test scores. The effect of the covariate on Test III was statistically removed, and the adjusted mean scores were assessed for both the treatment group (M = 58.62, SE = 2.01), and the control group (M = 46.38, SE = 2.13). The analysis was followed by the between-group test, which explained that after adjusting for pre-test scores, there was a significant difference between the *i*-CAM group and the conventional group on statistics performance, F(1, 50) = 17.507, p<0.01. The effect size considered with partial eta squared value was 0.27, which indicated a large effect size. It means that 27% of the variance in the scores of statistics performance on Test III was explained by the follow-up treatment in the treatment group.

To find out whether the combination of these differences among the three tests was large enough to be considered statistically significant after controlling for the pre-test scores, a multivariate analysis of covariance, or MANCOVA, test was conducted to analyse the results (Table 2). Preliminary data analysis was conducted to check and ensure that there were no violations of the assumptions of normality, univariate and multivariate outlier, linearity, homogeneity of variances, homogeneity of variance-covariance matrix, homogeneity of regression slopes, multicollinearity, and singularity with a reliable measurement of the pre-test as a covariate. This one-way multivariate analysis of covariance was performed on three dependent variables associated with the statistics performance: descriptive statistics, comparing means, and measures of association. An adjustment was made for one covariate, pre-statistics performance. The conducted test assessed the effect of the *i*-CAM in comparison with conventional instruction in terms of students’ scores on different statistics performance tests, depending on the topics (Test I, II, and III). The students’ scores on the pre-test administration of the statistics test were used as the covariate in this analysis.

**Table 2:** Multivariate Statistics Tests for Statistics Problem-Solving Performance.

Effect	Value	F(3, 45)	Sig.	Partial Eta Squared
**Interaction Group and Pre-test**	.959	.645	.590	.041
**Group**	.702	6.354	.001	.298

Table 2 shows that there was no significant relationship between the groups on the pre-test F(3, 45) = 0.645, p >0.05. Therefore, using the Wilks’ criterion, it could be concluded that the combined dependent variables were significantly related to the covariates, approximate F(3, 45) = 6.354, p < .05. Therefore, after adjusting for pre-test scores, there was a significant difference in the performance of the two groups, the *i*-CAM group and the conventional group, as indicated by the three tests on statistics performance (Tests I, II, and III). In addition, the partial eta squared value of almost 0.30, which was considered as a large effect size, showed that 29.8% of the covariance in test scores on the statistics performance, was explained by the treatment.

The results in Table 3 suggest that students in the treatment and control groups perform differently on their statistics-performance tests. The treatment group showed a more substantial increase in each of the statistics tests after participating in the three phases of treatment based on the *i*-CAM. On the other hand, the students in the control group appeared to get less benefit from simply having access to the materials in the *i*-CAM.

**Table 3:** Pairwise Comparisons of Multivariate Analysis.

Dependent Variable	Group		Mean Difference (I-J)	Std. Error	Sig.	Post Hoc
**Test I**	(I)Treatment	(J)Control	11.385	2.699	.000	(I) > (J)
**Test II**	(I)Treatment	(J)Control	9.553	3.954	.020	(I) > (J)
**Test III**	(I)Treatment	(J)Control	12.246	2.927	.000	(I) > (J)

## Discussion and Conclusion

For a long time, apprenticeship has been a part of learning [14]. It will probably never go away due to its rich history of actual use in helping a novice to become an expert. Noticeably, several theoretical perspectives such as social constructivism, ZPD, and situated learning provide a clear foundation to support cognitive apprenticeship efforts in maximizing learning and ensuring strong cognitive and social interactions, as well as many other significant instructional outcomes. In this study, the *i*-CAM group and the conventional group had the same lecturer and the same class duration (twelve weeks), as well as similar learning contents and assignments. The only difference between the two groups was how the learning activities were carried out. The *i*-CAM group was supported by Internet-based cognitive apprenticeship in which the expert monitor led the students in learning activities. The learning activities for the conventional group, on the other hand, consisted mainly of students going to the learning portal to use the learning materials, as well as having discussions with other peers after the face-to-face classroom lectures.

As stated previously, the core interest was in improving the students’ statistics problem-solving performance. Kuhs and Ball [35] declared that mathematics should be taught with a stress on the students’ performance. Accordingly, one of the goals of the development aspect of this study was to increase the technology capacity and competency of LMSs to improve learning performance. The results of the three post-tests showed that students in the treatment group obtained higher scores in statistics performance than did those in the control group. The mean scores of statistics performance significantly increased in the treatment group at each of the tests—Test I, Test II, and Test III—as compared to the mean scores for the control group, after adjusting for the pre-test scores. Many researchers [11, 36] also stated that exploring and applying the model of cognitive apprenticeship could facilitate students’ learning through embedded activities in social contexts with appropriation of a shared cognitive method.

The first phase of the treatment was coaching and modelling, and was also known as the handling phase. The students in the treatment group demonstrated higher statistics performance scores in Test I, II, and III compared to the control group. The Post Hoc test (Table 3) showed that there was a significant difference between the groups’ mean scores after adjusting for the pre-test scores. In fact, providing assistance by explicitly involving students, in the way that the experts planned, revised, and evaluated statistics problems and solutions, was effective for students in the treatment group within the asynchronous environment.

The results of this study were also in accordance with the result of a study in problem-solving effectiveness in the web-based cognitive apprenticeship model in facilitating 11- to 12-year-old students using the collaborative approach in a social science course [37]. The finding of this study was also consistent with a previous study [36], which showed that coaching was a remarkable approach where students strongly believed that coaching was useful in their mathematics learning. The first possible reason was that the Internet-based coaching-modelling provided clear and effective responses to the needs of the students. Brandt et al. [21] and Farmer et al. [22] introduced cognitive modelling as the heart of the cognitive apprenticeship model. Effective cognitive modelling and coaching referred to the expert who clearly described and showed his/her practical knowledge and thinking skills in dealing with complex tasks and in accordance to the learners’ needs [11, 18].

The next phase of treatment, or Phase II, focused on scaffolding, articulation, and reflection, and is also known as the supporting phase. Dabbagh [38] described promoting scaffolding and articulation as instructional strategies that embodied the pedagogical model for e-learning. In this study, at the end of this phase, the results of the students’ statistics performance as measured by Test II showed a significant difference between the treatment and control groups, after adjusting for pre-test scores. The possible reason for this difference was that the *i*-CAM in this phase provided assistance based on scaffolding for students in the way that the expert suggested and guided students during statistics problem-solving by providing effective hints to those in the treatment group within the asynchronous environment. Moreover, after fading the scaffolds, the monitor picked some of the complete answers and shared them; thus, the students had the opportunity to compare their own problem-solving process with those of their peers or monitor. These activities could engage students in the type of activities that analysed what they had written in the previous assignments, so they could make a judgment about their work and then try to apply the newly gained knowledge to revise their solutions [38].

In the last phase of treatment, also known as self-exploring, students followed up the treatment by using the discussion forum and interactions with peers. The results of Test III, carried out at the end of the phase, demonstrated a significant difference between the two groups, after adjusting for the pre-test scores. Since there were no interactions between students and the monitor in the treatment group, the module was the same for both groups. In this case, the reason behind this difference could be the effect of the instructional strategies that the *i*-CAM employed, which engaged the students in developing their knowledge and problem-solving skills individually within an asynchronous environment. The findings were supported by a previous study by Tsai et al. [39] in which they used a web-based CAM to improve students’ argumentation skills. Alger and Kopcha [20] also built and designed a technology-enhanced environment on an LMS based in cognitive apprenticeship, and their reported results indicated that the instructional methods based on the CAM were a useful and viable approach for acquiring skills.

In summary, the results could prove that learning under the CAM method of instruction engendered the positive changes of statistical performance among the experimental group. This finding is supported by social constructivism theories that clarify that students’ learning develops through collaboration with advanced peers’ or experts’ assistance [40]. It suggests that the more the systematic intervention framed on the CAM causes students to achieve, the more significant the outcomes. The results may stress the impact of cognitive apprenticeship even in an online environment. Many researchers also stated that exploring and applying the model of cognitive apprenticeship could facilitate students’ learning through embedded activities in social contexts with appropriation of a shared cognitive method. The results of this study may provide ideas and directions for the development and implementation of alternative teaching and learning strategies, especially in the extension of learning activities and communication in a virtual environment following the face-to-face sessions. It provides the advantages of online learning, while the students meet each other at the same place and at the same time every week. This is a vital investigation into the issue because many higher-learning institutions spend a large amount of money on maximizing the benefits of their e-learning operations.

This study assessed the efficacy of the *i*-CAM which was a model of contribution of components of Brown et al.’s model. It may provide an impetus to investigate more areas on employing the Internet-based cognitive apprenticeship model by using LMS in mathematics education. The findings of this study may also encourage other researchers to integrate the different contribution of these components and features to confirm these findings, particularly to determine which feature or contribution of features is responsible for the effects.

## Appendix 1

### Sample items on Tests

1. Which of the following constitute continuous variables?
  - Anxiety rated on a scale of 1 to 5 where 1 equals not anxious, 3 equals moderately anxious and 5 equals highly anxious
  - Height of buildings in meters
  - Education level (secondary schools certificate, colleges, diploma, universities bachelor, masters or Ph.D)
  - All of the above
2. You have measured emotional intelligence as a dependent variable. Which level of measurement have you collected?
  - Ratio
  - Interval
  - Ordinal
  - Nominal
3. Ratio data differs from interval data in that it has:
  - Greater precision
  - More possible scores
  - A fixed zero
  - All of the above
4. Which of the following are FALSE of descriptive statistics?
  - They can be used to describe independent variables or dependent variables
  - They look at numerical summaries variables
  - The numerical summaries can be used in comparing two or more groups
  - Frequency tables and frequency polygons are suitable for nominal data

22 What does “*p*” refer to in the analysis of data?
  - prove
  - significant
  - power
  - probability
23 Which of the following captures the meaning of the null hypothesis?
  - there is no relationships in the population
  - there is no relationships in the sample
  - there is no controls in the sample
  - there is no statistical tests
24 If a researcher wanted to test for differences across three means, which of the following test statistics would be most appropriate?
  - t
  - p
  - F
  - d
25 If a t-value was calculated to be 1.30, what would be its associated “*p*” when you write your conclusion?
  - >.05
  - <.05
  - <.01
  - <0.001

## Supporting Information

S1 Dataset The Entire Data Set Obtained in the Study. (XLSX)

S1 Fig Descriptive Analysis and Graphical Explanation of the Dataset. (a) Histogram with frequency curve for all test (pre-test, test I, test II, and test III) are shown. The frequency curve in the figures displayed that the scores in both groups appear to be reasonably normally distributed. (b) The normal Q-Q plot of the tests are displayed. The normality of the variable was also supported by an inspection of the normal probability plots or Normal Q-Q Plot. The figures showed that the observed value for each score was plotted against the expected value of the normal distribution. Hence, this reasonably straight line indicated a normal distribution.
(PDF)
