# Supplementary figures and images for: Effect of Internet-Based Cognitive Apprenticeship Model (i-CAM) on Statistics Learning among Postgraduate Students
Source: PLoS One. 2015 Jul 1;10(7):e0129938. doi: 10.1371/journal.pone.0129938 (PMC4488879; doi:10.1371/journal.pone.0129938)

**a.**

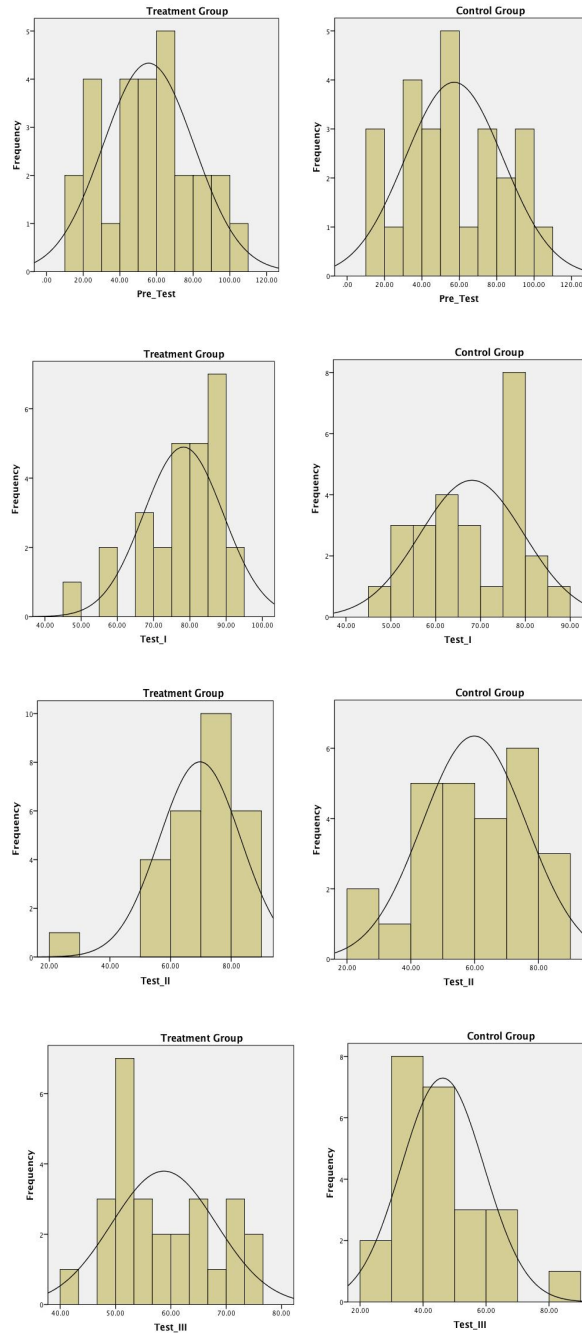

**b.**

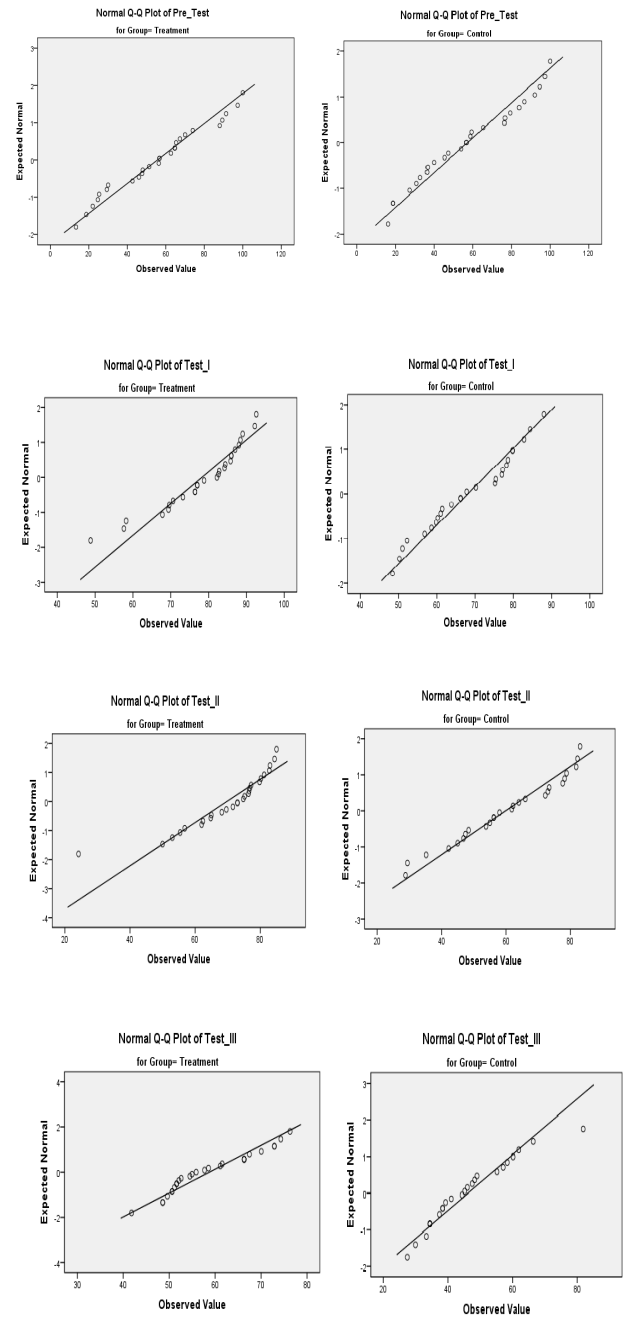

Supplement: S1 Fig — (a) Histogram with frequency curve for all test (pre-test, test I, test II, and test III) are shown. The frequency curve in the figures displayed that the scores in both groups appear to be reasonably normally distributed. (b) The normal Q-Q plot of the tests are displayed. The normality of the variable was also supported by an inspection of the normal probability plots or Normal Q-Q Plot. The figures showed that the observed value for each score was plotted against the expected value of the normal distribution. Hence, this reasonably straight line indicated a normal distribution. (PDF) [file pone.0129938.s002.pdf]
